# Characterization of *Plasmodium ovale curtisi* and *P. ovale wallikeri* in Western Kenya Utilizing a Novel Species-specific Real-time PCR Assay

**DOI:** 10.1371/journal.pntd.0003469

**Published:** 2015-01-15

**Authors:** Robin H. Miller, Clifford O. Obuya, Elizabeth W. Wanja, Bernhards Ogutu, John Waitumbi, Shirley Luckhart, V. Ann Stewart

**Affiliations:** 1 Preventive Medicine and Biometrics, Uniformed Services University, Bethesda, Maryland, United States of America; 2 Kondele Laboratory, U.S. Army Medical Research Unit-Kenya, Kisumu, Kenya; 3 Kondele Laboratory, USAMRU-K, Kisumu, Kenya; 4 Walter Reed Project, Kenya Medical Research Institute, Kisumu, Kenya; 5 Department of Medical Microbiology and Immunology, University of California Davis School of Medicine, Davis, California, United States of America; US Food and Drug Administration, UNITED STATES

## Abstract

**Background:**

*Plasmodium ovale* is comprised of two genetically distinct subspecies, *P. ovale curtisi* and *P. ovale wallikeri*. Although *P. ovale* subspecies are similar based on morphology and geographical distribution, allelic differences indicate that *P. ovale curtisi* and *P. ovale wallikeri* are genetically divergent. Additionally, potential clinical and latency duration differences between *P. ovale curtisi* and *P. ovale wallikeri* demonstrate the need for investigation into the contribution of this neglected malaria parasite to the global malaria burden.

**Methods:**

In order to detect all *P. ovale* subspecies simultaneously, we developed an inclusive *P. ovale*-specific real-time PCR assay based on conserved regions between *P. ovale curtisi* and *P. ovale wallikeri* in the reticulocyte binding protein 2 (rbp2) gene. Additionally, we characterized the *P. ovale* subspecies prevalence from 22 asymptomatic malaria infections using multilocus genotyping to discriminate *P. ovale curtisi* and *P. ovale wallikeri*.

**Results:**

Our *P. ovale rbp2* qPCR assay validation experiments demonstrated a linear dynamic range from 6.25 *rbp2* plasmid copies/microliter to 100,000 rbp2 plasmid copies/microliter and a limit of detection of 1.5 *rbp2* plasmid copies/microliter. Specificity experiments showed the ability of the *rbp2* qPCR assay to detect low-levels of *P. ovale* in the presence of additional malaria parasite species, including *P. falciparum*, *P. vivax*, and *P. malariae*. We identified *P. ovale curtisi* and *P. ovale wallikeri* in Western Kenya by DNA sequencing of the tryptophan-rich antigen gene, the small subunit ribosomal RNA gene, and the *rbp2* gene.

**Conclusions:**

Our novel *P. ovale rbp2* qPCR assay detects *P. ovale curtisi* and *P. ovale wallikeri* simultaneously and can be utilized to characterize the prevalence, distribution, and burden of *P. ovale* in malaria endemic regions. Using multilocus genotyping, we also provided the first description of the prevalence of *P. ovale curtisi* and *P. ovale wallikeri* in Western Kenya, a region holoendemic for malaria transmission.

## Author Summary

Humans can be infected with five malaria parasite species: *Plasmodium falciparum, P. vivax, P. malariae, P. knowlesi*, and *P. ovale*. Although the vast majority of malaria morbidity and mortality worldwide can be attributed to *P. falciparum*, non-falciparum malaria parasites can also cause clinical disease. Researchers use nucleic acid based detection methods, such a polymerase chain reaction (PCR), to detect low-density malaria parasitemias that can evade microscopic detection. *P. ovale* was recently identified to exist as two subspecies, *P. ovale curtisi* and *P. ovale wallikeri*, that look identical but differ genetically. In this study, we developed a novel real-time PCR (qPCR) assay to detect all *P. ovale* parasites, based on a conserved gene between *P. ovale curtisi* and *P. ovale wallikeri*. We also used DNA sequencing to differentiate between *P. ovale curtisi and P. ovale wallikeri* from a small sample of *P. ovale* asymptomatic infections in Western Kenya. Through the use of our novel *rbp2* qPCR assay, we aim to characterize the prevalence of *P. ovale* in future epidemiological studies in order to better understand this neglected malaria parasite species.

## Introduction

*Plasmodium ovale*, the causative agent of benign tertian malaria, was identified as a distinct malaria parasite species in 1922 based on its characteristic oval morphology in infected erythrocytes [1]. *P. ovale* rarely causes severe disease in humans living in malaria endemic regions, but can cause serious clinical disease in naive travelers [2–9]. The actual prevalence and clinical relevance of *P. ovale* is likely underestimated for the following reasons. First, *P. ovale* is often found as a mixed infection with other malaria parasite species [10–12]. This can confound microscopic identification of *P. ovale* due to difficulties in differentiating *P. ovale* from other morphologically similar malaria parasites, such as *P. vivax*. Second, the characteristic low-level parasitemia of *P. ovale* infection further complicates microscopic detection due to the difficulty in finding and identifying low numbers of *P. ovale* parasites [13]. Finally, malaria Rapid Diagnostic Tests (RDTs) show a reduced ability to detect *P. ovale* compared to other human malaria parasites, resulting in false negative cases [14–16]. However, the use of extremely sensitive molecular detection methods, such as polymerase chain reaction (PCR), have revealed a higher prevalence of *P. ovale* and expanded the geographical distribution of this malaria parasite compared to what was previously identified based on microscopy [10, 17–20].

Recent findings demonstrated that *P. ovale* exists as two genetically distinct sympatric subspecies, *P. ovale curtisi* and *P. ovale wallikeri*[21–24]. Morphological differences between the two *P. ovale* subspecies have not been identified, thereby limiting the use of microscopy to differentiate *P. ovale curtisi* and *P. ovale wallikeri*. As recent studies suggest potential clinical and latency duration differences between the two *P. ovale* subspecies, [25, 26], a discriminatory assay to differentiate *P. ovale curtisi* and *P. ovale wallikeri* is clinically relevant. Additionally, initial *P. ovale*-specific assays developed by our group and others were unknowingly designed based on gene sequences specific to only one subspecies, thereby failing to detect the other *P. ovale* subspecies. PCR assays that target conserved genetic regions between the two subspecies are, therefore, necessary to determine the true *P. ovale* prevalence and distribution [27–30].

Small-subunit ribosomal RNA (ssrRNA) genes are common targets for malaria parasite species-specific assays based on nucleotide polymorphisms that facilitate specific detection of the species of interest [28, 29, 31]. Although rRNA based PCR assays have proven useful for the detection of low-level parasitemias of a single malaria parasite species, Demas et al. demonstrated that alternative gene targets may be more sensitive for species-specific detection in the context of mixed species infections [32]. A quality control program to determine the ability of 10 different laboratories to detect malaria parasite species based on rRNA PCR revealed detection of *P. ovale* to be the most difficult, with a detection rate of 70% [33]. Additionally, allelic diversity within the *P. ovale* ssrRNA alleles may further limit the ability of rRNA specific PCR assays to detect *P. ovale* infections [34]. Due to these difficulties in the detection of *P. ovale*, we designed a novel *P. ovale*-specific assay based on a gene found only in *P. ovale curtisi* and *P. ovale wallikeri* and not present in other human malaria parasite species. This approach reduces aberrant amplification of non-target malaria species and allows for the detection of low-level *P. ovale* infections in the presence of high parasitemias of other malaria parasite species, such as *P. falciparum*.

Several epidemiology surveys of exant malaria species have established the endemicity of *P. ovale* in Western Kenya based on microscopic identification, entomological studies, and nucleic acid detection methods [13, 35–38]. Clinical cases due to *P. ovale* relapse in non-immune individuals after traveling to Western Kenya have also been reported, including a single case of a returned traveller with *P. ovale curtisi* infection [25, 39]. However, the lack of data on the prevalence and distribution of *P. ovale curtisi* and *P. ovale wallikeri* in Western Kenya represents a critical gap in our understanding of the true malaria epidemiology in this region that could impact both patient treatment and malaria control strategies.

In this study, we developed a novel, highly specific, real-time PCR (qPCR) assay to detect all *P. ovale* subspecies simultaneously based on a conserved region of the *P. ovale*-specific reticulocyte binding protein 2 (*rbp*2)gene. This inclusive *P. ovale rbp2* qPCR assay was characterized and validated to determine the sensitivity, limit of detection, limit of quantification, specificity, repeatability, and reproducibility. In addition, the occurrence of both *P. ovale* subspecies (*P. ovale curtisi* and *P. ovale wallikeri)* was documented in Western Kenya using multilocus genotyping. Our *P. ovale* species-specific assay can be utilized to better characterize the presence, parasitemia, geographical distribution, and the contribution of this malaria parasite species to mixed species infections and to clinical disease in malaria endemic regions.

## Methods

### Sample collection

Anonymized human whole blood samples were collected with signed informed consent under approved protocols (Walter Reed Army Institute of Research Human Use and Review Committee Protocols #1720 and 1306, Kenya Medical Research Institute (KEMRI) SSC#2008 and 1111). Clinically healthy (asymptomatic) adult individuals in Nyanza Province, Kenya were screened (active detection) with the Parascreen Pan/Pf ® malaria Rapid Diagnostic Test (Zephyr Biomedicals, Verna, Goa, India) for the presence/absence of malaria parasites from March through September of 2008. Thin and thick smears were examined subsequently by up to 5 expert microscopists in the Malaria Diagnostic Centre (MDC), Kisumu, Kenya for malaria species designation and estimation of quantitative parasitemia [40]. Samples identified as positive for *P. ovale* (n = 22) via microscopy, in which all were mixed infections with other malaria species, were targeted for DNA extraction and PCR based analysis. DNA was extracted from 200 microliters of whole blood using the QIAamp DNA Minikit (Qiagen, Venlo, Netherlands) following the manufacturer’s protocol. DNA was eluted in 200 microliters of Buffer EB and samples were stored at −20°C until time of use. A human-specific RNaseP based qPCR assay was performed for each sample in duplicate to confirm successful nucleic acid extraction [41].

### Characterization of *P. ovale* subspecies in Western Kenya

**Tryptophan-rich antigen (*tra*) gene**. The *P. ovale*-specific tryptophan-rich antigen (*tra*) gene was recently identified as a target to discriminate between *P. ovale* subspecies based on DNA sequence length and single nucleotide polymorphisms (SNPs) [22, 23, 30]. We utilized the PoTRA fwd3 and PoTRA rev3 primers reported in Oguike et al. 2011 for PCR analysis [23]. Primers (Table 1) were synthesized by Integrated DNA Technologies (IDT, Coralville, IA, USA) and purified by standard desalting methods. Each PCR assay consisted of 1X Sigma JumpStart REDTaq ReadyMix (20 mM Tris-HCl, 100 mM KCl, 4 mM MgCl_2_, 0.4 mM of each dNTP, 0.03 unit/μl of Taq DNA polymerase, Sigma, Balcatta, WA, USA), 8.75 picomoles of each primer, and one microliter of template with a final volume of 25 microliters. PCR cycling conditions were: initial denaturation for 2 minutes at 95°C followed by 45 cycles of 95°C for 30 seconds, 58°C for 45 seconds, 72°C for 1 min and a final extension at 75°C for 5 minutes. All conventional PCRs were performed on a DNA Engine PTC-200 Thermal Cycler (MJ Research, Waltham, MA, USA).

**Table 1:** Primer and probe sequences utilized for conventional PCR and qPCR experiments.

**Target**	**Primer/Probe Name**	**Primer/Probe Sequence**	**Reference**
**Tryptophan-rich antigen (*tra)***	PoTRAfwd3	5’-GCACAAAAATGGTGCTAACC-3’	Oguike et.al 2011 [23]
	PoTRArev3	5’-ATCCATTTACCTTCCATTGC-3’	
**Small subunit rRNA (ssrRNA)**	rOVA1WC	5’-TGTAGTATTCAAACGCAGT-3’	Fuehrer et.al 2012 [29]
	rOVA2WC	5’-TATGTACTTGTTAAGCCTTT-3’	
**Reticulocyte binding protein 2 (*rbp2*)**	PoRBP2fwd1	5’-CCACAGATAAGAAGTCTCAAGTACGATATT-3’	
	PoRBP2rev1	5‘-TTGGAGCACTTTTGTTTGCAA-3’	
	PoRBP2p	5’-6FAM-TGAATTGCTAAGCGATATC-MGB-3’	

**Figure 1:**
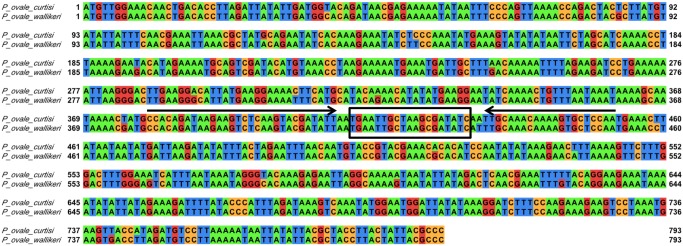
*P. ovale* reticulocyte binding protein 2 (*rbp2*) sequence alignment. The *P. ovale curtisi* (GU813971) and *P. ovale wallikeri* (GU813972) *rbp2* sequences were aligned using EMBL-EBI Clustal Omega program and visualized in Jalview with the default Jalview nucleotide color scheme (green for adenine, orange for cytosine, red for guanine, and blue for thymine). Primers and probe were designed based on conserved regions between the two *P. ovale* subspecies. The forward (PoRBP2fwd1) and reverse (PoRBP2rev1) primers are indicated by arrows and the hydrolysis probe (PoRBP2p) binding site (boxed) is located in between the forward and reverse primer.

**Reticulocyte binding protein 2 (*rbp2*) gene**. The reticulocyte binding protein 2 (*rbp2)* gene was utilized by Oguike et al. 2011 to differentiate between *P. ovale* subspecies using qPCR melt curve profiles based on six SNPs present within a 120 base pair fragment. We designed a novel set of primers (Table 1, IDT) using Primer Express software (Life Technologies, version 3.0; Frederick, MD, USA) to amplify a smaller, 74 base pair region of the *rbp2* gene for assay development. Our primers (PoRBP2f and PoRBP2r) are located within conserved DNA sequences of the *P. ovale* subspecies to ensure detection and amplification of both *P. ovale* subspecies. The amplicon also contains a single SNP to distinguish *P. ovale* subspecies by DNA sequencing. Fig. 1 shows the single SNP in the *rbp2* amplicon at position 431, in which *P. ovale curtisi* contains an adenine and *P. ovale wallikeri* contains a thymine. Primer BLAST was utilized to ensure our primers were specific for *P. ovale* and would not amplify non- *P. ovale* malaria parasite DNA or human DNA. PCRs consisted of 1X Sigma JumpStart REDTaq ReadyMix Reaction Mix, 25 picomoles of each primer, and one microliter of template, with a final volume of 25 microliters. PCR cycling conditions were as follows: initial denaturation at 95°C for 2 minutes followed by 40 cycles of 95°C for 30 seconds, 55°C for 30 seconds, 72°C for 30 seconds, and a final extension at 72°C for 10 minutes.

**Small subunit ribosomal RNA (ssrRNA) gene**. We utilized *P. ovale*-specific primers (Table 1, IDT) reported by Fuehrer et al. 2012 (rOVA1WC and rOVA2WC) to further characterize *P. ovale* positive samples based on differences within the small subunit ribosomal RNA (ssrRNA) gene [29]. PCRs consisted of 1X Sigma JumpStart REDTaq ReadyMix Reaction Mix, 25 picomoles of each primer, one microliter of template, and a final volume of 25 microliters. PCR cycling conditions were as follows: initial denaturation at 95°C for 4 minutes followed by 35 cycles of 94°C for 1 minute, 58°C for 2 minutes, 72°C for 2 minutes, and a final extension at 72°C for 5 minutes.

**DNA sequencing**. PCR products were visualized on 0.7% agarose gels stained with ethidium bromide. PCR products were cloned into the pCR 2.1-TOPO TA vector (Life Technologies) based on manufacturer’s guidelines. Plasmid purification was performed using the QIAprep Spin Miniprep kit (Qiagen) and used as template for sequencing reactions. PCR products were sequenced using the M13 Forward (−20) Primer (Life Technologies) at the Biomedical Instrumentation Center at the Uniformed Services University or GENEWIZ Inc (Germantown, MD, USA) using the ABI 3500XL Genetic Analyzer and the ABI 3730XL DNA Analyzer, respectively. Sequencing facility was chosen based on temporal availability. DNA sequences were aligned and analyzed with previously published sequences using SeqMan software (DNAStar Lasergene Version 8.1.5, Madison, WI, USA). Reference sequences utilized for DNA alignments are shown in Table 2.

**Table 2:** GenBank accession numbers used for DNA alignment of the *P. ovale curtisi* and *P. ovale wallikeri tra, rbp2*, and ssrRNA DNA sequences.

**Target**	**Reference Sequence**	**GenBank Accession Number**
**Tryptophan-rich antigen (*tra)***	*P. ovale curtisi* type 1	HM594182
	*P. ovale curtisi* type 2	HM594183
	*P. ovale wallikeri* type 1	HM594180
	*P. ovale wallikeri* type 2	HM594181
**Small subunit rRNA (ssrRNA)**	*P. ovale curtisi*	JF894405
	*P. ovale wallikeri*	JF894406
**Reticulocyte binding protein 2 (*rbp2)***	*P. ovale curtisi*	GU813971
	*P. ovale wallikeri*	GU813972

### Real-time PCR assay to detect *P. ovale*

Primer Express software (Life Technologies, version 3.0) was utilized to design a hydrolysis probe (Table 1) for use with our *rbp2* primers on the ABI 7500 fast real-time PCR (qPCR) platform (Life Technologies). An alignment of the *P. ovale rbp2* DNA sequences was constructed using the Clustal Omega Program provided by the European Molecular Biology Laboratory—European Bioinformatics Institute (EMBL-EBI) [42, 43]. We utilized the Jalview output tool to visualize the DNA sequence alignment (Fig. 1)[44]. Primers and probe were designed in order to amplify a conserved region within the *rbp2* gene to ensure detection of both *P. ovale* subspecies by our qPCR assay at the same time. *In silico* analyses were performed to ensure primers and probe were specific to *P. ovale* and would not amplify genes of other malaria parasites or human DNA. Each qPCR reaction consisted of the following: 1X TaqMan Fast Universal PCR Master Mix, No AmpErase UNG (Life Technologies, Cat No. 4364103), 5 picomoles of each primer and probe, and one microliter of template in a final volume of 20 microliters. Real-time PCR was performed utilizing fast thermal cycling conditions (95°C for 20 seconds, followed by 40–60 cycles of 95°C for 3 seconds and 60°C for 30 seconds). Analysis of qPCR results was performed using ABI 7500 Fast Real-Time PCR Systems Software (Life Technologies, Version 2.0.5). Basic statistical analyses (means, standard deviations, coefficient of variation), generation of standard curve graphs, calculation of slopes, and coefficient of correlation were performed in Microsoft Excel or GraphPad Prism (GraphPad Prism Software Version 6, La Jolla, CA, USA).

**Plasmid standard curve**. We cloned the 74 base pair *rbp2* amplicon into the pCR 2.1-TOPO TA vector (Life Technologies) following manufacturer’s guidelines and eluted the *rbp2* plasmid in PCR grade water. The approximate *rbp2* amplicon copy number per microliter was determined based on spectrophotometer (Nanodrop 2000c) concentration in nanograms per microliter. Plasmids with the *rbp2* amplicon (*rbp2* plasmid) were diluted in water to generate a ten-fold serial dilution from 100,000 *rbp2* copies per microliter to 0.1 *rbp2* copies per microliter. The resulting non-linearized ten-fold serial dilution series was utilized as a standard curve in subsequent validation experiments including determination of the linear dynamic range, specificity, reproducibility, repeatability, and limit of detection. The effect of the conformation of the *rbp2* plasmid on standard curve linearity was analyzed by linearizing the *rbp2* plasmid using the NotI restriction enzyme (New England BioLabs Inc, Ipswich, MA, USA) according to the manufacturer’s protocol. *Rbp2* plasmid linearization was confirmed by gel electrophoresis on a 0.7% agarose gel stained with ethidium bromide. Linearized *rbp2* plasmid was purified using the Qiagen PCR Purification Kit following the manufacturer’s protocol. The approximate *rbp2* copy number per microliter of the linearized *rbp2* plasmid was determined and diluted in water to generate a ten-fold serial dilution (100,000 to 0.1 copies per microliter). The *rbp2* standard curve PCR efficiency and coefficient of correlation (R^2^) were determined and the Pearson product-moment correlation was used to compare the linearized and non-linearized *rbp2* plasmid standard curves (GraphPad Prism).

**Validation experiments**. Real-time PCR efficiency was determined using a standard curve of 10-fold serial dilutions of the non-linearized *rbp2* plasmid. Efficiency (E) was calculated using the following formula: *E* = 10^(− 1/ *slope*)^ −1. *Rbp2* plasmid standard curve samples were run at least in duplicate and the mean quantification cycle (Cq) value was utilized to generate the standard curve. The limit of detection was defined as the concentration of *rbp2* plasmid in copies per microliter that gave a positive signal in at least one replicate well in two separate qPCR experiments. Limit of quantification was defined as the range of *rbp2* plasmid concentrations that maintained linearity and therefore could be used to quantify *P. ovale* concentration from test samples.

Specificity was analyzed using DNA template from non- *P. ovale* malaria parasite species and uninfected human DNA. Genomic DNAs from *P. falciparum* strains 3D7 (WRAIR), FCR3CSA (ATCC/BEI Resources, MR4, Manassas, Virginia), Dd2 (ATCC/BEI Resources, MR4), and NF54 (ATCC/BEI Resources, MR4) were utilized as template to assess specificity. *P. vivax* genomic DNA was extracted from frozen whole parasites (kind gift of Dr. J. Prachumsri, Mahidol University, Bangkok, Thailand). Since pure *P. malariae* positive samples were unavailable, we utilized three samples collected as part of the blood collection protocol in Kenya that were positive for *P. malariae* as well as *P. falciparum* by microscopy and PCR, but were negative for *P. ovale*. The *P. malariae* parasitemias ranged from approximately 30 to 2400 parasites per microliter. Additionally, genomic DNAs from *P. knowlesi, P. simiovale, P. fragile*, and *P. cynomolgi* (ATCC/BEI Resources, MR4), were also utilized as templates. Specificity was further analyzed by performing spiking experiments in which a known concentration of *rbp2* plasmid was added to template containing *P. falciparum* 3D7 DNA (10,000 parasites per microliter) or *P. vivax* DNA (517 parasites per microliter). One-way analysis of variance (ANOVA) was used to determine differences in Cq values for spiking experiments (GraphPad Prism).

Within-run repeatability was defined as the variation of Cq values within a single run and was analyzed by calculating the percent coefficient of variation (%CV) of Cq values in replicate wells. Between-run repeatability was defined as the variation of Cq values in separate qPCR runs and was determined by calculating the percent coefficient of variation (%CV) of mean Cq values based on six separate qPCR experiments. Reproducibility was evaluated by comparing the assay performance by a technician at the USAMRU-K laboratory in Kisumu, Kenya and the Uniformed Services University in Bethesda, Maryland, USA.

**Quantification comparison: Microscopy versus *rbp2* qPCR**. Parasitemias were determined for *P. ovale* positive blood films based using standard microscopic methods at the Malaria Diagnostic Centre, affiliated with both USAMRU-K and KEMRI, in Kisumu, Kenya. DNA was extracted from microscopy-positive *P. ovale* samples and tested using the *P. ovale*-specific *rbp2* qPCR assay. Approximate *rbp2* copy number per microliter was determined based on the *rbp2* plasmid standard curve. Parasitemias as determined by expert microscopy (parasites per microliter) were compared to *rbp2* copy number per microliter as determined by the *P. ovale*-specific qPCR in order to examine potential correlation between *rbp2* plasmid copy number and microscopic parasitemias.

## Results

### *P. ovale* subspecies characterization

**Human-specific RNaseP qPCR**. A previously described qPCR assay based on the human-specific RNaseP gene was performed to confirm the presence of nucleic after DNA extraction [41]. The human RNaseP gene was detected from all 22 samples (Average Cq = 29.12, Cq Range = 28.2–32.87, standard deviation = 1.02), indicating extraction methods yielded DNA suitable for subsequent PCR experiments.

**Tryptophan-rich antigen (*tra*) gene**. Alignments of *tra* gene sequences revealed nine samples (40.9%) positive for *P. ovale curtisi* type 1, two samples (9.1%) positive for *P. ovale curtisi* type 2, six samples (27.3%) positive for *P. ovale wallikeri* type 1, and three samples (13.6%) positive for *P. ovale wallikeri* type 2 (Table 3). Previously published GenBank accession numbers were utilized as reference sequences for alignment and are shown in Table 2. Representative *P. ovale curtisi* type 1, *P. ovale curtisi* type 2, *P. ovale wallikeri* type 1, and *P. ovale wallikeri* type 2 *tra* DNA sequences were deposited under GenBank accession numbers KM494978-KM494981, respectively, and are identical to the reference sequences. As shown in Table 4, unique polymorphisms within the *tra* gene were also detected and confirmed by at least two separate sequencing reactions for 5 samples: Po05, Po12, Po20, Po06, and Po07 (Accession numbers KM494982-KM494986, respectively). Samples Po12 and Po20 contained an 18 base pair insertion between nucleotide positions 171 and 172 (based on *P. ovale wallikeri* type 1 HM594180 reference sequence), which represents a short sequence repeated throughout the *tra* gene. Two samples, Po9 and Po18, failed to amplify with the *tra* primers despite multiple PCR attempts.

**Table 3:** *P. ovale* subspecies identification by DNA sequencing of the of the tryptophan-rich antigen *(tra)* gene, the reticulocyte binding protein 2 (*rbp2*) gene, and the small subunit ribosomal RNA (ssrRNA) gene.

**Sample ID**	**Co-infecting malaria species (parasites/μl)**	***P. ovale*** microscopy (parasites/μl)	**Tryptophan-rich antigen (*tra*)**	**Reticulocyte binding protein 2 (*rbp2*)**	**Small subunit rRNA (ssrRNA)**
**Po1**	*P. falciparum* (7334) *P. malariae* (110.8)	57.6	*P. ovale curtisi* type 1	*P. ovale curtisi*	*P. ovale curtisi*
**Po2**	*P. falciparum*(653.4) *P. malariae* (114)	156	*P. ovale curtisi* type 1	*P. ovale curtisi*	*P. ovale curtisi*
**Po3**	*P. falciparum* (67121.1)	458	*P. ovale curtisi* type 2	*P. ovale curtisi*	*P. ovale curtisi*
**Po4**	*P. falciparum* (571.5) *P. malariae* (56)	42	*P. ovale wallikeri* type 2	*P. ovale wallikeri*	*P. ovale wallikeri*
**Po5**	*P. falciparum* (101.8)	121.78	*P. ovale wallikeri* type 2	*P. ovale wallikeri*	*P. ovale wallikeri*
**Po6**	*P. falciparum* (306.1) *P. malariae* (1320)	2321.78	*P. ovale curtisi* type 1	*P. ovale curtisi*	*P. ovale curtisi*
**Po7**	*P. falciparum* (3284.2) *P. malariae* (1648.6)	69.33	*P. ovale curtisi* type 1	*P. ovale curtisi*	*P. ovale curtisi*
**Po8**	*P. falciparum* (4568) *P. malariae* (320)	296.35	*P. ovale curtisi* type 1	*P. ovale curtisi*	*P. ovale curtisi*
**Po9**	*P. falciparum* (515) *P. malariae* (255.3)	16	No data	*P. ovale curtisi*	No data
**Po10**	*P. falciparum* (1897.3)	456.89	*P. ovale curtisi* type 2	*P. ovale curtisi*	*P. ovale curtisi*
**Po11**	*P. falciparum* (412.7) *P. malariae* (583.3)	16	*P. ovale curtisi* type 1	*P. ovale curtisi*	No data
**Po12**	*P. falciparum* (158.9) *P. malariae* (48)	331.26	*P. ovale wallikeri* type 1	*P. ovale wallikeri*	*P. ovale wallikeri*
**Po13**	*P. falciparum* (613.8) *P. malariae* (453.1)	365.54	*P. ovale curtisi* type 1	*P. ovale curtisi*	*P. ovale curtisi*
**Po14**	*P. falciparum* (16703.3) *P. malariae* (32)	157.33	*P. ovale wallikeri* type 1	*P. ovale wallikeri*	*P. ovale wallikeri*
**Po15**	*P. falciparum* (28976)	3738.88	*P. ovale wallikeri* type 1	*P. ovale wallikeri*	*P. ovale wallikeri*
**Po16**	*P. falciparum* (3889.9) *P. malariae* (211.8)	32	*P. ovale wallikeri* type 2	*P. ovale wallikeri*	*P. ovale wallikeri*
**Po17**	*P. falciparum* (16) *P. malariae* (24)	1118.08	*P. ovale curtisi* type 1	*P. ovale curtisi*	*P. ovale curtisi*
**Po18**	*P. falciparum* (382.1) *P. malariae* (52.4)	26.67	No data	*P. ovale curtisi*	No data
**Po19**	*P. falciparum* (8954.2) *P. malariae* (409.6)	58.67	*P. ovale curtisi* type 1	*P. ovale curtisi*	*P. ovale curtisi*
**Po20**	*P. falciparum* (197.3) *P. malariae* (304)	350.61	*P. ovale wallikeri* type 1	*P. ovale wallikeri*	*P. ovale wallikeri*
**Po21**	*P. falciparum* (4299.5) *P. malariae* (172)	84.36	*P. ovale wallikeri* type 1	*P. ovale wallikeri*	*P. ovale wallikeri*
**KSI**	*P.falciparum* (no data)	No data	*P. ovale wallikeri* type 1	*P. ovale wallikeri*	*P. ovale wallikeri*

**Table 4:** Five *P. ovale* positive samples contained unique *tra* gene polymorphisms identified by DNA sequencing.

**Nucleotide Position (Genbank accession number)**	**71**	**99**	**171–172**	**523**	**595**
***P. ovale wallikeri*** type 1 (HM594180)^a^	T	G	-^b^	T	G
**Po05 (KM494982)**	C	G	-^b^	C	AA
**Po12 (KM494983)**	T	C	ATAAATGCTATAACCCCC	T	G
**Po20 (KM494984)**	T	C	ATAAATGCTATAACCCCC	T	G
**Nucleotide Position**	**280**	**307**	**664**		
***P. ovale curtisi*** type 1 (HM594182)^c^	A	A	G		
**Po06 (KM494985)**	G	A	A		
**Po07 (KM494986)**	A	G	G		

^a^ *P. ovale wallikeri* type 1 (HM594180, nucleotides 1–1171) was utilized as a reference for DNA sequence alignment of *P. ovale wallikeri* positive samples with unique polymorphisms (Po05, Po12, and Po20).

^b^ Dashes (-) indicate lack of an insertion. Samples Po12 and Po20 contained an 18 base pair insertion between nucleotide position 171 and 172 based on the reference sequence.

^c^ *P. ovale curtisi* type 1 (HM594182, nucleotides 1–1117) was utilized as a reference for DNA sequence alignment of *P. ovale curtisi* positive samples with unique polymorphisms (Po06 and Po07).

**Reticulocyte binding protein 2 (*rbp2*) gene**. DNA sequences of the *rbp2* gene were obtained for all 22 *P. ovale* samples (Table 3). S1 Table contains the 74 pair *rbp2* amplicon for both *P. ovale curtisi* and *P. ovale wallikeri*. These sequences were not eligible for submission as the minimum length requirement for GenBank is 200 nucleotides. *P. ovale* subspecies results based on *rbp2* gene sequences agreed with subspecies results based on the *tra* gene sequences. Thirteen (59%) of the *P. ovale* samples were positive for *P. ovale curtisi* and 9 (41%) were positive for *P. ovale wallikeri*. None of our samples failed to amplify with the *rbp2* primers.

**Small subunit rRNA (ssrRNA) gene**. Nineteen of the 22 *P. ovale* positive samples were detected by the ssrRNA gene assay (Table 3). *P. ovale curtisi* and *P. ovale wallikeri* ssrRNA sequences were approximately 99% identical to previously published sequences at this locus. Representative *P. ovale curtisi* and *P. ovale wallikeri* ssrRNA sequences were deposited in GenBank as KM494987 and KM494988, respectively. *P. ovale* subspecies results based ssrRNA gene sequences agreed with subspecies results based on *tra* and *rbp2* gene sequences. Three samples, Po9, Po11, and Po18, failed to amplify using the ssrRNA primers despite a second attempt using an additional microliter of template DNA.

### Real-time PCR to detect *P. ovale*

**Figure 2:**
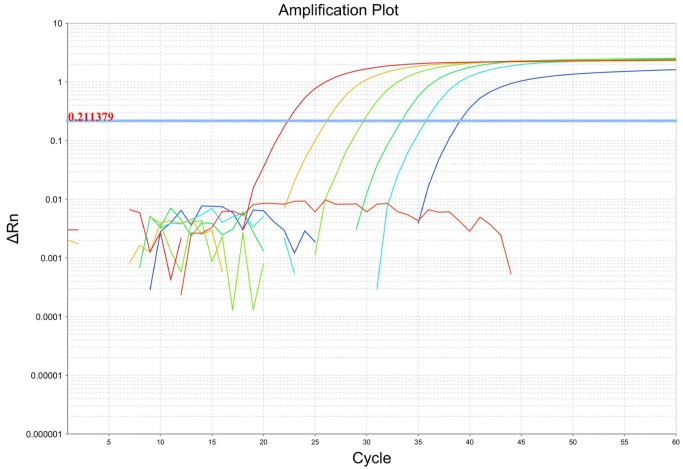
*P. ovale rbp2* qPCR dynamic range. A ten-fold serial dilution of *rbp2* plasmid (1 to 100,000 copies/μl) is shown in the amplification plot. The cycle threshold was determined automatically by the ABI 7500 fast system software program. The negative control sample (red line) shows no amplification over the cycle threshold for 60 cycles.

**Figure 3:**
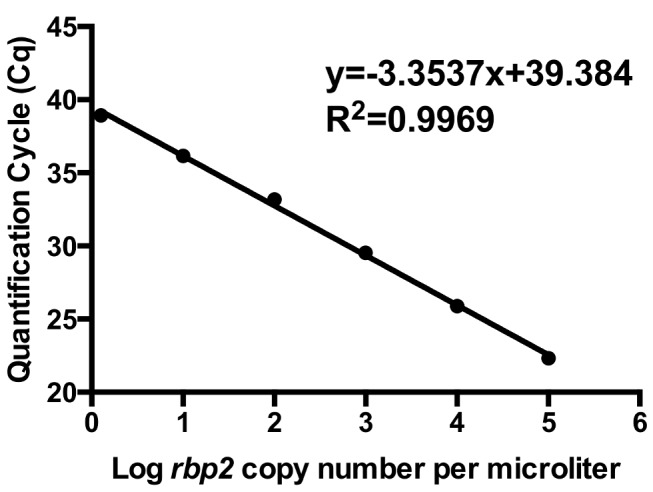
*P. ovale rbp2* plasmid standard curve. A representative standard curve demonstrates linearity based on 10-fold serial dilutions (1 to 100,000 copies/μl) of *rbp2* plasmid.

**Plasmid standard curve analysis of rbp2 qPCR assay**. Since all 22 *P. ovale* microscopy positive samples were successfully amplified and sequenced using the *rbp2* primers, we developed an *rbp2* based qPCR assay to detect all *P. ovale* subspecies simultaneously in a single assay. Efficiency of the *rbp2* qPCR assay was analyzed using the non-linearized *rbp2* plasmid 10-fold serial dilution standard curve. Efficiency ranged from 90%–99% for six consecutive qPCR experiments with a coefficient of correlation (R^2^) greater than 0.99. A representative qPCR amplification plot and standard curve are shown in Fig. 2 and 3, respectively. All 22 *P. ovale* samples identified as *P. ovale* positive by expert microscopy were detected using our *rbp2* qPCR assay. There was no difference in PCR efficiency or R^2^ value based on the conformation (linearized vs. non-linearized) of the *rbp2* plasmid standard curve (Pearson product-moment correlation = 0.998, P<0.001).

**Limit of quantification and limit of detection**. The linear dynamic range of the *rbp2* qPCR assay was determined to be between 6.25 copies per microliter and 100,000 copies per microliter based on serial dilutions of the *rbp2* plasmid. Two-fold serial dilutions of known concentrations of the *rbp2* plasmid were performed in at least duplicate to determine the limit of detection. Dilutions containing 1.5 copies per microliter of the *rbp2* plasmid were detected by at least one replicate well in two separate qPCR experiments.

**Specificity**. In order to test the specificity of our *rbp2* assay for *P. ovale*, we performed qPCR using DNA isolated from cultured *P. falciparum* 3D7 (10,000 parasites per microliter) and *P. vivax* DNA (517 parasites per microliter). Based on a series of ten separate qPCR experiments, DNA from *P. falciparum* and *P. vivax* were uniformly negative. To ensure no background from other *P. falciparum* strains, we tested genomic DNAs from strains Dd2, NF54, and FCR3CSA, which were also not detected by our assay. We tested DNA from *P. knowlesi, P. fragile*, and *P. cynomolgi* and found DNA from these malaria parasite species were undetectable by our *rbp2* qPCR assay. As we were unable to obtain pure *P. malariae* samples, we examined DNA samples isolated from the blood of individuals co-infected with both *P. malariae* and *P. falciparum*. These *P. falciparum* and *P. malariae* co-infected samples were also negative, indicating that our *rbp2* qPCR assay does not detect *P. malariae* DNA. Two different control DNA samples from malaria uninfected human blood were also uniformly negative. All specificity experiments were carried out to 60 cycles in an attempt to capture non-specific amplification, which was never seen, although the standard curve and the *P. ovale*-containing field samples amplified appropriately.

**Figure 4:**
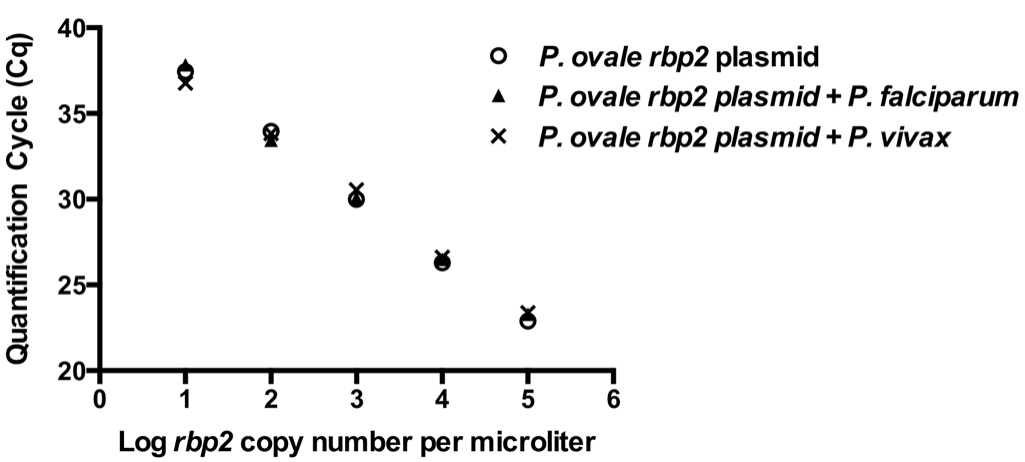
*P. ovale rbp2* qPCR specificity. Serial dilutions of *rbp2* plasmid were spiked with *P. falciparum* DNA (10,000 parasites/μl) or *P. vivax* DNA (517 parasites/μl). Cq values were unchanged in the presence of DNA from additional malaria parasite species (P = 0.9993).

Spiking experiments, in which *P. falciparum* DNA or *P. vivax* DNA was added to the *rbp2* plasmid standard curve samples and subsequently utilized as template for the *rbp2* qPCR did not significantly alter the Cq values compared to when the standard curve plasmid samples were run alone (ANOVA, P = 0.9993, Fig. 4).

Interestingly, our *rbp2* qPCR assay detected *P. simiovale* genomic DNA isolated from filter paper. DNA sequencing utilizing the *rbp2* primers revealed that the 74 base pair *rbp2* region in *P. simiovale* is identical to that in *P. ovale curtisi*. Subsequent attempts using additional primers to sequence the full-length *rbp2* gene of *P. simiovale* were not successful. As these additional primers successfully amplified *P. ovale* positive samples, the inability to amplify the full-length *P. simiovale rbp2* gene is likely due to sequence polymorphisms between *P. ovale* and *P. simiovale* in the primer binding regions.

**Repeatability**. Within-run repeatability of the *rbp2* plasmid standard curve Cq values was high, with the percent coefficient of variation (%CV) of dilution series replicates between 0.00–2.23% (Table 5). Results were also repeatable between runs, with the percent coefficient of variation (%CV) between 1.17–3.43% (Table 5). Repeatability was determined using results from six separate consecutive qPCR experiments.

**Table 5:** Repeatability and reproducibility of the *rbp2* plasmid standard curves determined via Cq values from six separate qPCR experiments.

***Rbp2* plasmid Copies/μl**	**100,000**	**10,000**	**1,000**	**100**	**10**
**Within-run Repeatability (%CV)**	0.25–0.74	0.41–1.00	0.048–1.47	0.00–1.52	0.19–2.23
**Between-run repeatability (%CV)**	2.21	1.53	1.17	1.46	3.43

**Reproducibility**. Analysis of the efficiency of the *rbp2* assay was performed independently at the USAMRU-K laboratory. A known concentration of non-linearized *rbp2* plasmid was diluted in PCR grade water to generate a 10-fold dilution standard curve for PCR efficiency analysis. The assay was performed with the same *P. ovale*-specific primers and probe utilized in validation experiments in a final volume of 50 microliters of Life Technologies TaqMan Fast Master Mix for the USAMRU-K ABI 7500. Despite slight variations in qPCR set up and cycling conditions, the Kenya laboratory obtained a PCR efficiency of 93.6% with an R^2^ >0.99 for the standard curve analysis. These results are identical to the PCR efficiencies and R^2^ values obtained at USU. The USAMRU-K laboratory also performed specificity experiments and demonstrated no amplification from *P. falciparum* DNA, DNA from uninfected human blood, or from negative template controls.

**Figure 5:**
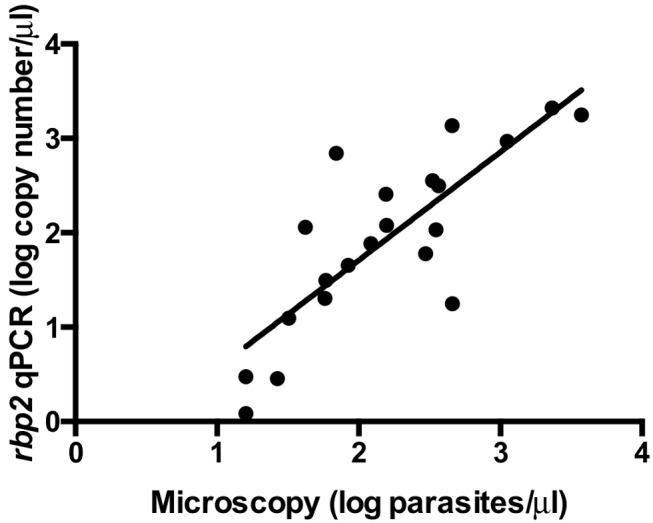
Comparison of microscopy and *P. ovale rbp2* qPCR results. *P. ovale* parasitemias based on microscopy (log parasites/μl) were compared to *rbp2* qPCR results (log *rbp2* copy number/μl). A limited correlation was found between parasitemia and *rbp2* plasmid copy number (R2 = 0.6595).

**Quantification comparison: Microscopy versus *rbp2* qPCR**. Quantitative parasitemia determined by expert microscopy (parasites per microliter) was compared to the *rbp2* copy number per microliter based on the *rbp2* plasmid standard curve (Fig. 5). A modest correlation was determined (R^2^ = 0.6595). This lack of a strong correlation is not surprising, as all *P. ovale* parasitemias were low, ranging from 16–3800 parasites/μl, and such low-level parasitemias are notoriously difficult to quantify accurately by microscopy [40, 45–47]. Additionally, the samples utilized for comparison were mixed malaria species infections, mainly with *P. falciparum*. Mixed species infections create further difficulties for the accurate quantification of *P. ovale*-specific parasitemia based on light microscopy, but single-species *P. ovale* infected samples were not available.

## Discussion

Based on multilocus genotyping using the *rbp2*, ssrRNA, and *tra* genes, we detected both *P. ovale curtisi* and *P. ovale wallikeri* in approximately equal frequencies in a small sample set from Western Kenya, a region in which *P. ovale* subspecies characterization had not been previously performed. The presence of both *P. ovale* subspecies in Western Kenya is in agreement with other studies in sub-Saharan Africa and *P. ovale* endemic regions that describe the sympatric distribution of *P. ovale curtisi* and *P. ovale wallikeri* [23, 27, 48]. We also identified additional allelic diversity within the *tra* gene in *P. ovale* samples from Kenya (Table 4) compared to what was previously identified in *P. ovale* samples from other malaria endemic regions [23]. This allelic diversity at the *P. ovale tra* gene is consistent with reports of other *tra* variants identified by DNA sequencing, however our *tra* sequences are unique from previously published *tra* gene sequences [30].

Our new inclusive *P. ovale*-specific qPCR assay is based on *rbp2*, a gene that contains conserved regions between *P. ovale curtisi* and *P. ovale wallikeri* but that is absent from other human malaria parasite species. The *rbp2* qPCR assay described herein allows simultaneous detection of both *P. ovale* subspecies using a single set of primers and probe. All 22 samples were detected and sequenced using our *rbp2* primers, highlighting the utility of these primers for *P. ovale* identification. *P. ovale* subspecies differentiation by DNA sequencing of the 74 base pair *rbp2* sequence region was in absolute agreement with *tra* and ssrRNA DNA sequencing results. This again emphasizes the utility of the PoRBP2fwd1 and PoRBP2rev1 primers for *P. ovale* subspecies discrimination based on a single SNP at position 431 (Fig. 1) located between these primers. In agreement with other previous studies, these data demonstrate perfect dimorphism between *P. ovale curtisi* and *P. ovale wallikeri*, providing further support for the separation of the two *P. ovale* subspecies [21–24, 48–50]. As we begin to understand potential clinical, pathological, and biological differences between the two *P. ovale* subspecies, molecular methods to distinguish *P. ovale curtisi* and *P. ovale wallikeri* will aid in these research efforts. Additionally, as genomic data and full genome sequences become available for *P. ovale curtisi* and *P. ovale wallikeri*, phylogenetic analyses to determine the evolutionary relatedness between these and other malaria species will likely further our understanding of these newly characterized but poorly understood human parasites.

Using the *rbp2* plasmid as a standard curve, the linear dynamic range of our assay was determined to be between 6.25 copies of *rbp2* per microliter to 100,000 copies of *rbp2* per microliter. The lower, non-linear but still clearly positive limit of detection of our assay was determined to be 1.5 copies of *rbp2* per microliter, confirming this assay’s capacity to detect low-level parasitemias. *P. ovale* parasitemias are characteristically lower than other malaria species, so we limited the testing of our upper dynamic range to 100,000 *rbp2* copies per microliter, as higher copy numbers would likely be epidemiologically and clinically irrelevant. We used the *rbp2* plasmid to determine the linear dynamic range and limit of detection because of difficulties obtaining pure *P. ovale* infected samples from malaria endemic regions and the inability to culture *P. ovale* parasites. The paucity of published genomic information for *P. ovale* also hinders the determination of copy number of *P. ovale*-specific genes, such as the *rbp2, tra*, and ssrRNA genes, utilized in this study. Thus, we are further limited in our attempts to appropriately correlate *rbp2* copy number and *P. ovale* parasitemias. Despite these limitations, we demonstrate the utility of our *P. ovale*-specific assay to detect low-levels of the *rbp2* plasmid and to detect low *P. ovale* parasitemias (as low as 16 parasites per microliter) from human blood samples collected in Western Kenya. Our study was also limited by only testing samples collected in Western Kenya and additional validation is therefore needed to confirm the ability of the *rbp2* qPCR assay to detect total *P. ovale* from other malaria endemic regions. As the 22 samples included in this study were identified as *P. ovale* by microscopy, further studies are needed to test the *P. ovale rbp2* qPCR assay with submicroscopic and asymptomatic *P. ovale* infections with a range of parasitemias.

Repeatability and reproducibility of qPCR assays are important components of assay validation as they indicate the assay’s capacity to provide consistent and reliable results in different environments. Different users under modified laboratory conditions performed this assay successfully, with high PCR efficiency and equivalent quantification.

Specificity experiments showed no cross reactivity of our assay with *P. falciparum, P. vivax*, *P. malariae, P. cynomolgi, P. knowlesi, P. fragile*, and DNA from uninfected human blood even when qPCR was performed for 60 cycles. The complete lack of background amplification from human and other malaria parasite DNA, verifies the exquisite specificity of the assay. Further, assay performance was unchanged in the presence of DNA from other malaria parasite species. This is of particular importance for *P. ovale*, as this malaria species is often found as a co-infection with other malaria species. Interestingly, our *rbp2* qPCR assay also detected DNA obtained from *P. simiovale*. As *P. simiovale rbp2* sequence information is not available, we attempted to amplify the full-length *P. simiovale rbp2* gene using additional primers based on the *P. ovale rbp2* gene. However, we were unable to amplify the full *P. simiovale rbp2* gene, suggesting the *P. ovale* and *P. simiovale rbp2* genes may be similar but not identical. These results warrant further investigation of the *P. simiovale rbp2* and additional specificity experiments of other *P.ovale* assays that may also unknowingly detect *P. simiovale*.

Of the 22 samples identified as *P. ovale* positive by expert microscopy, three samples (Po9, Po11, Po18) failed to amplify at two of the three loci tested despite multiple attempts (Table 3). However, the *rbp2* gene was successfully amplified for all 22 samples as was a human-specific RNaseP endogenous control. These data, along with the parasitemia data from multiple expert microscopists, indicate that the 22 samples were *P. ovale* positive and that DNA template quality was unlikely to be the cause of the failed amplifications at the *tra* and ssrRNA loci. The inability to successfully amplify at all three loci could be explained by several reasons including: sequence polymorphisms, template degradation, low *P. ovale* density, and inter-laboratory variability due to reagents, equipment, and personnel. Additional investigation into potential reasons for the failure to amplify at all loci was prevented due to limited sample volume.

The limited correlation between microscopy and *rbp2* qPCR results (Fig. 5) is not surprising as parasitemia calculations for *P. ovale* human samples at low parasitemias are notoriously difficult, particularly in co-infected samples [45]. Our *P. ovale* positive samples from Western Kenya are all co-infected with either *P. falciparum* or *P. malariae*, thus likely complicating the microscopy quantitation further. Variation between parasitemia and *rbp2* copy number could also be explained by the *P. ovale* parasite stage. For example, a *P. ovale* ring stage counts as a single parasite by microscopy and DNA extracted from a *P. ovale* ring stage parasite represents one genome. However, a *P. ovale* schizont is counted as a single parasite by microscopy but DNA extracted from a *P. ovale* schizont may contain up to 14 genomes. This is a limitation of our study, as any relationship between *P. ovale* parasitemia and *rbp2* copy number based on qPCR would depend on the parasite stages observed under the microscope and present in the blood sample obtained for DNA extraction.

Utilizing a plasmid standard curve for qPCR assays provides an efficient method for standardizing assays that does not require culturing organisms or using human samples. However, recent studies have highlighted important concerns regarding the plasmid template conformation that could lead to quantification bias of plasmid template by qPCR [51, 52]. After linearizing our template plasmid to compare with a non-linearized plasmid standard curve, we found no difference in Cq value, R^2^, slope, or PCR efficiency with the *rbp2* qPCR assay. This is in agreement with another recent study, which also found no difference in plasmid standard curve based on the plasmid confirmation (linearized versus non-linearized) [53]. These results indicate that the effect of plasmid conformation on standard curve quantification may be assay specific. In addition to plasmid conformation, several additional quality control factors were optimized, including plasmid isolation methods, purification, storage, and developing appropriate laboratory protocols to minimize freeze-thawing, handling, and contamination.

Conventional PCR assays targeting the multi-copy small subunit ssrRNA genes are sensitive methods to detect and differentiate malaria species [54]. Initial *P. ovale*-specific ssrRNA PCR protocols showed limited capability to detect both *P. ovale* subspecies and have since been adapted to target conserved regions between the two subspecies. [29, 55, 56]. Although ssrRNA conventional PCR protocols have shown high sensitivity and specificity for malaria detection, we aimed to develop a novel *P. ovale*-specific assay based on a gene target that is found only in *P. ovale* and is absent from other malaria species infecting humans. We believe this approach enhances the specificity of our *P. ovale*-specific assay and eliminates the potential for nonspecific amplification of non- *P. ovale* species. Additionally, allelic variation within the ssrRNA genes of *P. ovale curtisi* and *P. ovale wallikeri* may limit the ability of ssrRNA based assays to capture all *P. ovale* infections due to sequence polymorphisms [34]. We found no allelic variation in the primer and probe-binding regions of the *rbp2* gene from 22 *P. ovale* positive samples, indicating the potential utility of *rbp2* for *P. ovale* subspecies detection.

While nested PCR is often utilized to enhance sensitivity for malaria PCR detection, we chose a single step qPCR protocol, as a nested PCR approach requires additional labor and cost to perform the second PCR. Nested PCR also increases the risk of laboratory contamination of PCR product and requires separate laboratory space to minimize the risk of contamination. Our *P. ovale*-specific qPCR assay maintains high sensitivity while also minimizing the additional cost, labor, designated laboratory space, and potential PCR product contamination that can be associated with nested PCR protocols.

Our *P. ovale*-specific qPCR assay provides several advantages for our future epidemiological studies of this neglected, and clinically relevant, malaria parasite species. First, fast qPCR conditions allow for a reaction to be completed in less than 1 hour. Second, the qPCR platform bypasses the need for gel electrophoresis, reducing the risk of amplicon contamination of the laboratory. Third, the use of a hydrolysis probe increases specificity compared to double stand DNA (dsDNA) based qPCR product detection. Our *P. ovale*-specific *rbp2* qPCR assay can be utilized to better characterize the presence, parasitemia, geographical distribution, and the contribution of *P. ovale* to mixed-species infections and to clinical disease in malaria endemic regions.

## Supporting Information

S1 Table *P. ovale curtisi* and *P. ovale wallikeri* reticulocyte binding protein 2 (*rbp2*) DNA sequences. (PDF)
