# Supplementary material for: Characterization of Plasmodium ovale curtisi and P. ovale wallikeri in Western Kenya Utilizing a Novel Species-specific Real-time PCR Assay
Source: PLoS Negl Trop Dis. 2015 Jan 15;9(1):e0003469. doi: 10.1371/journal.pntd.0003469 (PMC4295880; doi:10.1371/journal.pntd.0003469)
Supplement: S1 Table — (PDF) [file pntd.0003469.s001.pdf]

***P. ovale* subspecies**	***Rbp2* DNA sequences (5’-3’)**
P. ovale curtisi	CCACAGATAAGAAGTCTCAAGTACGATATTAATGAATTGCTAAGCGAT ATCAATTGCAAACAAAAGTGCTCCAA
P. ovale wallikeri	CCACAGATAAGAAGTCTCAAGTACGATATTAATGAATTGCTAAGCGAT ATCATTTGCAAACAAAAGTGCTCCAA
